# Supplementary figures and images for: First report of the ectomycorrhizal fungal community associated with two herbaceous plants in Inner Mongolia, China
Source: PeerJ. 2023 Jul 14;11:e15626. doi: 10.7717/peerj.15626 (PMC10351511; doi:10.7717/peerj.15626)

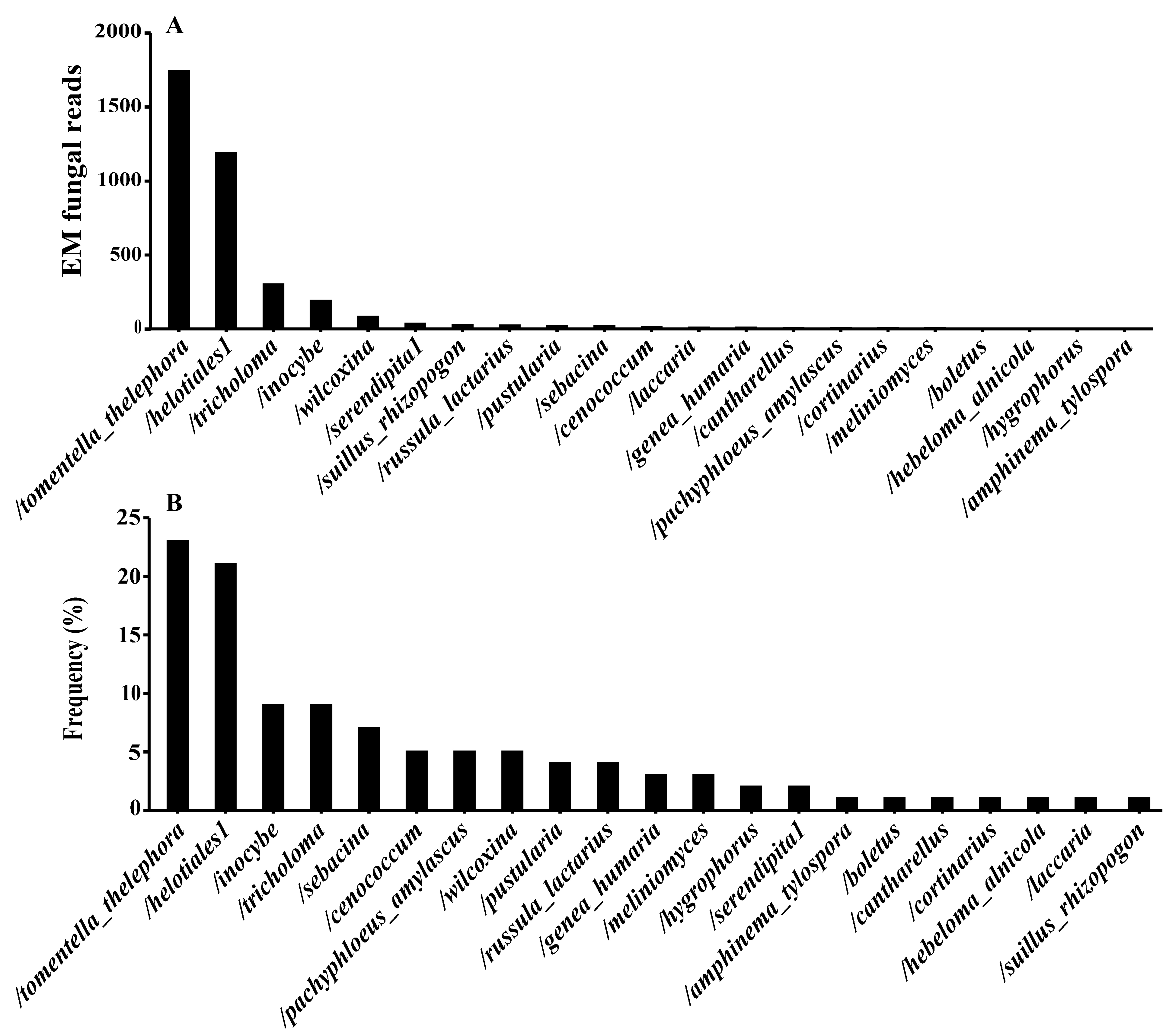

Supplement: Supplemental Information 5 [file peerj-11-15626-s005.pdf]

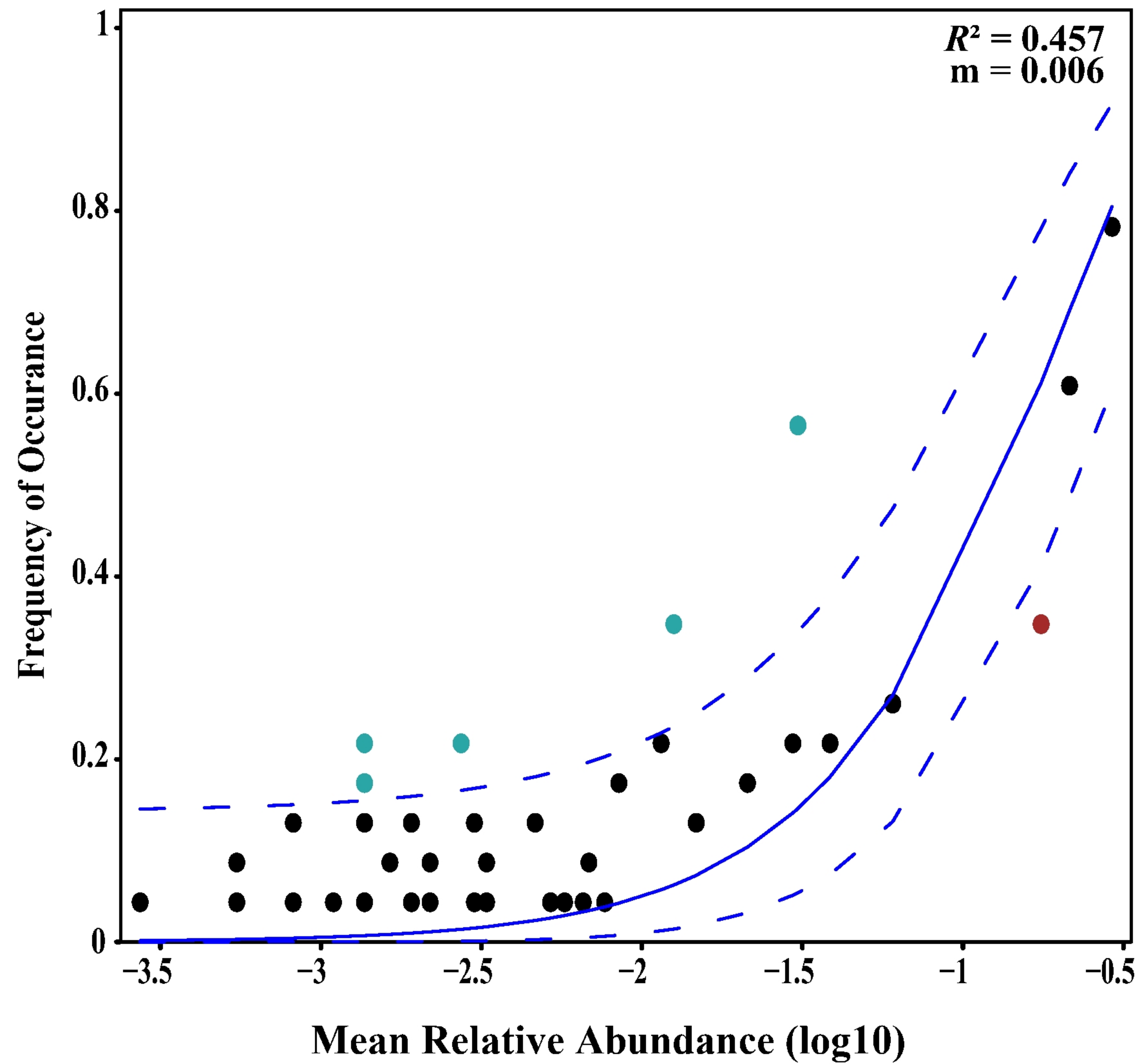

Supplement: Supplemental Information 6 — The neutral community model (NCM) of community assembly for EM fungal. [file peerj-11-15626-s006.pdf]
